# Surgical Outcomes of Aldosterone-Producing Adenoma on the Basis of the Histopathological Findings

**DOI:** 10.3389/fendo.2021.663096

**Published:** 2021-09-06

**Authors:** Huiping Wang, Fen Wang, Yushi Zhang, Jin Wen, Dexin Dong, Xiaoyan Chang, Hao Sun, Xiaosen Ma, Yunying Cui, Shi Chen, Lin Lu, Weidong Ren, Anli Tong, Yuxiu Li

**Affiliations:** ^1^Department of Endocrinology, Key Laboratory of Endocrinology, National Health Commission of the People’s Republic of China, Peking Union Medical College Hospital, Peking Union Medical College, Chinese Academy of Medical Sciences, Beijing, China; ^2^Department of Endocrinology, The First Affiliated Hospital of North University of Hebei, North University of Hebei, Zhangjiakou, China; ^3^Department of Endocrinology, Tongji Hospital, Tongji Medical College, Huazhong University of Science and Technology, Wuhan, China; ^4^Department of Urology, Peking Union Medical College Hospital, Peking Union Medical College, Chinese Academy of Medical Sciences, Beijing, China; ^5^Department of Pathology, Peking Union Medical College Hospital, Peking Union Medical College, Chinese Academy of Medical Sciences, Beijing, China; ^6^Department of Radiology, Peking Union Medical College Hospital, Peking Union Medical College, Chinese Academy of Medical Sciences, Beijing, China

**Keywords:** aldosterone-producing adenoma (APA), computed tomography, hypokalemia, surgical outcomes, CYP11B2 (aldosterone synthase), immunohistochemistry (IHC)

## Abstract

**Introduction:**

Previous studies on the surgical outcomes of aldosterone-producing adenoma (APA) patients were mainly based on the histopathological diagnosis of HE staining or adrenal venous sampling (AVS) instead of the functional pathology. The aim of the present study was to evaluate the surgical outcomes of APA patients based on the functional pathological diagnosis of APA according to HISTALDO (histopathology of primary aldosteronism) consensus.

**Methods:**

Clinical data of 137 patients with suspected APA were analyzed retrospectively. All patients had hypertension and spontaneous hypokalemia. In all patients, CT showed a unilateral solitary hypodense adrenal lesion, and a contralateral adrenal gland of normal morphology. Tumors were removed and immunostained for CYP11B2, and their pathology were identified based on HISTALDO consensus. Patients were followed up 6 to 24 months after operation.

**Results:**

Among 137 cases of presumptive APA diagnosed by CT, 130 (95%) cases were pathologically diagnosed with classical pathology, including 123 APA(90%) and 7 aldosterone-producing nodule (APN) (5%). 7 cases (5%) had non-functioning adenoma (NFA) with aldosterone-producing micronodule (APM) or multiple aldosterone-producing micronodule (MAPM) in the surrounding adrenal tissue. In all 137 patients, hypertension was complete or partial clinical success postoperatively. Complete clinical success was achieved in 73 (53%), and partial clinical success was achieved in 64 (47%) cases. Serum potassium level recovered to normal in all. In 123 patients with APA, complete clinical success was reached in 67 (54%), and partial clinical success was reached in 56 (46%) cases. Gender, duration of hypertension and the highest SBP were significant independent predictors for cure of APA after surgery. A multiple logistic regression model integrating the three predictors was constructed to predict the outcome, which achieved a sensitivity of 72.4% and a specificity of 73.1%.

**Conclusion:**

The specificity of CT in the diagnosis of APA and APN patients with hypokalemia was 95%. All patients achieved complete or partial clinical success after surgery. Gender, duration of hypertension and the highest SBP were independent predictors for the postoperative cure of APA.

## Introduction

Primary aldosteronism (PA) is a common cause of secondary hypertension, with its prevalence standing at about 5-19% (1). PA can be categorized into two major subtypes: unilateral aldosterone-producing adenoma (APA), accounting for about 30%, and bilateral adrenal hyperplasia (BAH), making up about 60% of all PA patients (2). Other less common types are unilateral adrenal hyperplasia (UAH), familial hyperaldosteronism, adrenocortical carcinoma, ectopic tumor, *etc.* PA can be divided into unilateral lesions and bilateral lesions according to the diseased side. Unilateral lesions are mostly seen in unilateral APA and UAH, and bilateral ones in BAH. Unilateral adrenalectomy can cure unilateral disease, whereas pharmacotherapy is the main strategy for bilateral disease (3). Therefore, it is important to distinguish unilateral from bilateral PA.

Adrenal vein sampling (AVS) is at present deemed as the gold standard for the classification of PA. The sensitivity and specificity of AVS for detecting unilateral aldosterone excess are 95% and 100% respectively (3, 4). However, AVS is invasive and brings radiation exposure to patients. In addition, although AVS can distinguish between unilateral and bilateral disease, unilateral aldosterone hypersecretion is not necessarily APA. Unilateral single or multiple APA, UAH and asymmetric BAH all can present lateralized aldosterone secretion on AVS (5–7). In contrast, adrenal computed tomography (CT) is simple and noninvasive, and can directly show adrenal lesions. However, CT may misdiagnose nonfunctional adenoma (NFA) as APA or miss small APA. The sensitivity and specificity of CT for the diagnosis of unilateral disease, with AVS as gold standard, were 78% and 75%, respectively (3, 4, 8, 9).

Histopathology with hematoxylin and eosin (HE) staining cannot distinguish NFA from APA. In 2014, a mouse monoclonal antibody against aldosterone synthase (CYP11B2) was established, which made it possible to study the function of adrenal adenoma (10). In 2021,HISTALDO (histopathology of primary aldosteronism) consensus for unilateral PA, based on HE staining and CYP11B2 immunohistochemistry (IHC), had been achieved (11).

Previous studies on the surgical outcome of APA patients were mainly based on the histopathology of HE staining or AVS (7, 12–14). NFA could be misdiagnosed as APA by HE staining, while lateralization on AVS, indicative of APA, could not exclude UAH or BAH with lateralization. In both cases, the surgical remission rate of APA might well be underestimated.

The aim of the present study was to evaluate the surgical outcome of APA patients based on functional pathological diagnosis of APA according to HISTALDO consensus.

## Methods and Materials

### Patients

We studied 137 patients with PA who underwent tumor resection at Peking Union Medical College Hospital between 2016 and 2019. All patients had clinical hypertension and spontaneous hypokalemia. Hypokalemia was defined as serum potassium level lower than 3.5 mmol/L according to its normal range in our hospital, which is also widely accepted cut-off. In all patients, there was no history of gastrointestinal potassium loss such as diarrhea and vomiting, and no history of use of thiazides and loop diuretics or liquorice. All had both CT imaging and histopathology. In all the patients, their plasma renin activity (PRA) was <1 ng/ml/h, plasma aldosterone concentration (PAC) >12 ng/dl (measured by radioimmunoassay), and ALD to PRA ratio (ARR)>30. Fifth-three patients had a PAC over 20 ng/dl plus PRA below detection levels, and were diagnosed as PA according to PA guideline (15). These patients did not need further confirmatory testing. In the other 84 patients, 45 patients were confirmed to be PA by Captopril suppression test, and 39 patients did not undergo confirmatory testing. These 39 patients were suspected as PA preoperatively and were confirmed by histopathology. Cushing syndrome was excluded in all the patients by measuring plasma adrenocorticotropic hormone (ACTH) level, serum cortisol level, and urinary free cortisol excretion.

The following scan parameters of CT were used: 120 kVp; variable tube current with automatic tube current modulation activated; collimation =128*0.6 mm; rotation time=0.28 s; filter kernel=B30f (medium smooth); pitch=0.9; reconstructed slice thickness and intervals=3 mm. Theoretically, a tumor with size larger than 3 mm could be detected on CT scan. A tumor with size larger than 6 mm, which can be found in at least two slices, definitely can not be missed. Diagnostic criteria of APA on adrenal CT scan was that it showed a unilateral solitary hypodense adrenal lesion with a CT value no higher than 20 which was consistent with the radiological feature of cortical adenoma, and a contralateral adrenal gland of normal morphology.

Written informed consent was obtained from each patient, and the study was approved by the Ethics Committee on Human Research of Peking Union Medical College Hospital, Beijing, China. Standard biosecurity and institutional safety procedures were adhered in the study.

Retrospectively studied were clinical data of the patients, including blood pressure and serum potassium, duration of hypertension, family history, history of diabetes and cardiovascular diseases, and laboratory results involving serum potassium, PAC, PRA, and imaging and pathological characteristics of the tumors.

Patients were followed up 6 to 24 months after operation. Blood pressure (including office and home blood pressure), serum potassium, antihypertensive medication and potassium supplementation were recorded. Complete clinical success and partial clinical success were defined according to PASO (16). Cure of hypokalemia was defined as serum potassium level over 3.5mmol/L without potassium supplementation.

### Immunohistochemistry and Pathological Classification

IHC of aldosterone synthase CYP11B2 was performed in tumor tissues, by using EnVision detection kit (Dako). Dilution of the monoclonal antibody against CYP11B2 was 1:200. The mouse monoclonal CYP11B2 antibody was kindly provided by Dr Celso E. Gomez-Sanchez (Department of Medicine, University of Mississippi Medical Center, Jackson, Mississippi).Adrenal cortical lesions were classified based on HE staining and CYP11B 2 immunostaining according to HISTALDO consensus. The histopathologic results were independently reviewed by two experienced pathologists.

### Statistical Analysis

All values were described as mean ± standard deviation or median (the 25th and 75th percentiles) for continuous variables with or without normal distribution, respectively. Student’s t-test of two independent samples was used for the data of normal distribution, and the Mann-Whitney test of two independent samples was used for the data of non-normal distribution. Chi-square test was employed for evaluating the association between two categorical variables. Binary logistic regression analysis was utilized to identify predictors of outcome of APA after surgery. The receiver operating characteristic (ROC) curve was used to assess the efficacy of each method and to establish a prediction model. Hosmer–Lemeshow test was performed to assess the goodness of fit of the logistic model. All the aforementioned statistical analyses were carried out by using SPSS 23 software package. Madcalc software was used to compare the areas under the ROC curve (AUC) between two groups. A P< 0.05 was considered statistically significant.

## Results

### Specificity of Adrenal CT Imaging in APA Diagnosis

**Figure 1:**
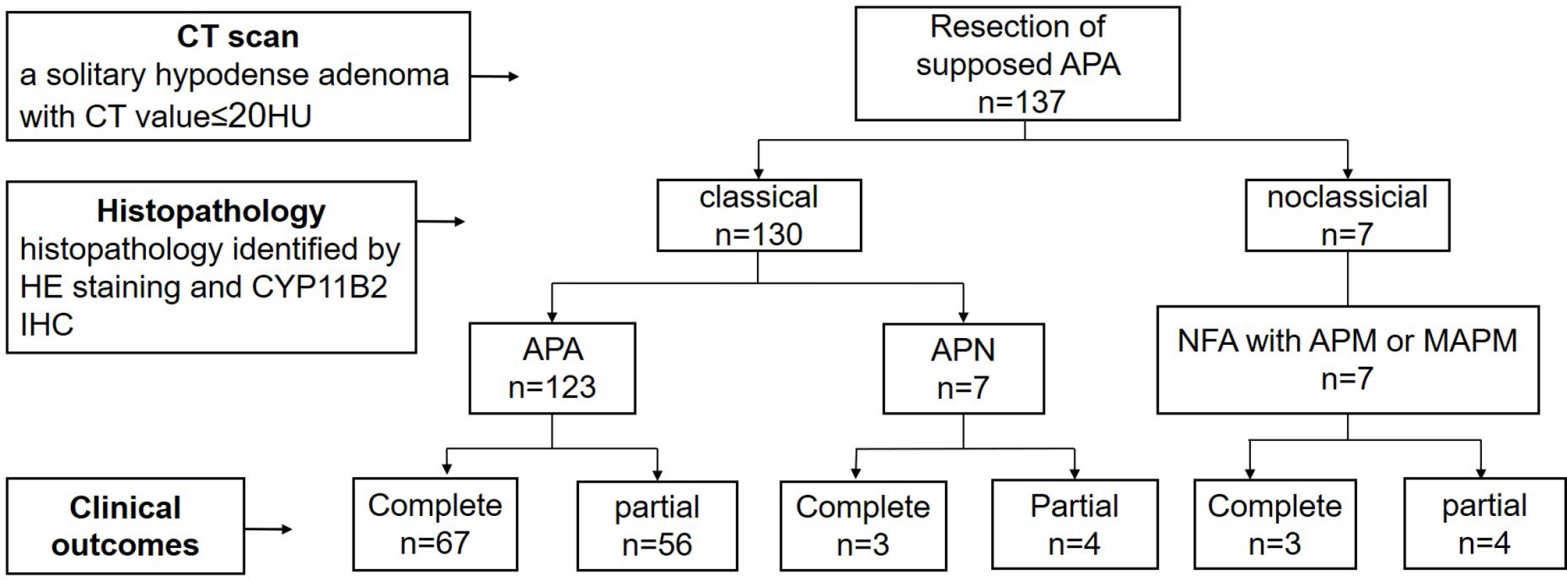
The procedure about preoperative diagnosis of APA, histopathology diagnosis and clinical outcomes. APA, aldosterone producing adenoma; APN, aldosterone-producing nodule; NFA, non-functional adenoma; APM, aldosterone-producing micronodule; MAPM, Multiple aldosterone-producing micronodules.

Among 137 cases of presumptive APA diagnosed by CT, 130 (95%) cases were pathologically diagnosed with classical PA, including 123 APA(90%) and 7 aldosterone-producing nodule (APN)(5%). 7 cases (5%) had non-functioning adenoma (NFA) with aldosterone-producing micronodule(APM) or multiple aldosterone-producing micronodule(MAPM) in the surrounding adrenal tissue, which attributed to nonclassical PA. Therefore, the specificity of CT imaging in the diagnosis of classical PA was 95% (**Figure 1**).

### Clinical Characteristics of PA

The clinical characteristics of 123 cases of APA, 7 cases of APN and 7 cases of nonclassical PA (NFA combined with AMP or MAPM) were compared. Duration of hypertension was different in the three groups. Nonclassical patients had the longest course of hypertension, followed by APA patients, and APN patients had the shortest duration. Compared with nonclassical patients, APA patients had a higher PAC and a lower LDL level. Blood pressure and serum potassium level did not show any difference among the three groups (**Table 1**). NFA patients were between 34 and 72 years old, and APA patients were between 21 and 69 years old. 34 years old was the cutoff to distinguish APA with 100% specificity.

**Table 1:** Clinical characteristics of patients with aldosterone-producing adenoma, aldosterone-producing nodule and non-functioning adenoma.

Variable	APA (N = 123)	APN (N = 7)	NFA with APM or MAPM (N = 7)
**Age (years)**	45 ± 10^&&^	47 ± 7	57 ± 14
**Gender (M/F)**	54/69	3/4	2/5
**Duration of hypertension (months)**	60 (24,120)*^&^	12 (6,32)^##^	126 (72,180)
**Duration of hypokalemia (months)**	6 (3,24)	6 (5,67)	19 (1,36)
**Preoperatively**			
**The highest SBP (mmHg)**	182 ± 23	189 ± 27	179 ± 21
**The highest DBP (mmHg)**	112 ± 13	108 ± 17	106 ± 10
**Serum potassium (mmol/L)**	2.5 ± 0.5	2.9 ± 0.3	2.8 ± 0.5
**PRA (ng/ml/h)**	0.01 (0.01,0.03)	0.01 (0.01,0.01)	0.02 (0.01,0.02)
**PAC (ng/dl)**	20.3 ± 7.0^&^	15.4 ± 3.6	14.4 ± 2.8
**LDL (mmol/L)**	2.59 ± 0.69^&&^	2.57 ± 0.71^#^	3.51 ± 0.65
**Family history of hypertension**	22% (27/122)	0% (0/7)	0% (0/7)
**Diabetes**	10% (12/120)	0% (0/7)	29% (2/7)
**Smoker**	16% (20/122)	29% (2/7)	29% (2/7)
**Cardiac complications**	34% (34/99)	25% (1/4)	33% (2/6)
**Renal complication**	0% (0/123)	0% (0/7)	0% (0/7)
**Tumor size (mm)**	17 (13,22)**	8 (7,9)^##^	23 (15,23)
**Tumor location (Left/Right)**	67/56^&^	5/2	7/0
**CTvalue (HU)**	2.0 (-5.0,7.3)	5.2 (-9.2,12.4)	3.3 (-5.2,5.1)
**Postoperatively**			
**SBP (mmHg)**	128 ± 12^&^	130 ± 10	140 ± 29
**DBP (mmHg)**	84 ± 8	83 ± 6	86 ± 12
**Serum potassium (mmol/L)**	4.2 ± 0.4	4.1 ± 0.3	4.0 ± 0.1
**Cure of hypertension**	54% (67/123)	43% (3/7)	43% (3/7)
**Antihypertensive medications**	28% (34/123)	57% (4/7)	57% (4/7)
**Potassium supplementation**	0% (0/123)	0% (0/7)	0% (0/7)

*P < 0.05 **P < 0.01 APA vs.APN; ^#^P < 0.05 ^##^P < 0.01 APN vs. NFA; ^&^P < 0.05 ^&&^P < 0.01 APA vs. NFA.

APA, aldosterone-producing adenoma; APN, aldosterone-producing nodule; NFA, non-functioning adenoma; MAPM, multiple aldosterone-producing micronodule; APM, aldosterone-producing micronodule; PRA, plasma renin activity; PAC, plasma aldosterone concentration.

### Surgical Outcomes

All 137 patients achieved complete or partial clinical success postoperatively. Seventy-three cases (53%) achieved complete clinical success, and 64 cases (47%) achieved partial clinical success. Serum potassium level recovered to normal in all. In APA patients, complete clinical success was achieved in 67 cases (54%),and partial clinical success in 56 cases (46%), while in APN patients and nonclassical PA patients, only 43% patients reached complete clinical success (**Figure 1** and **Table 1**).

### Factors Affecting Surgical Outcomes and the Prediction Model

**Figure 2:**
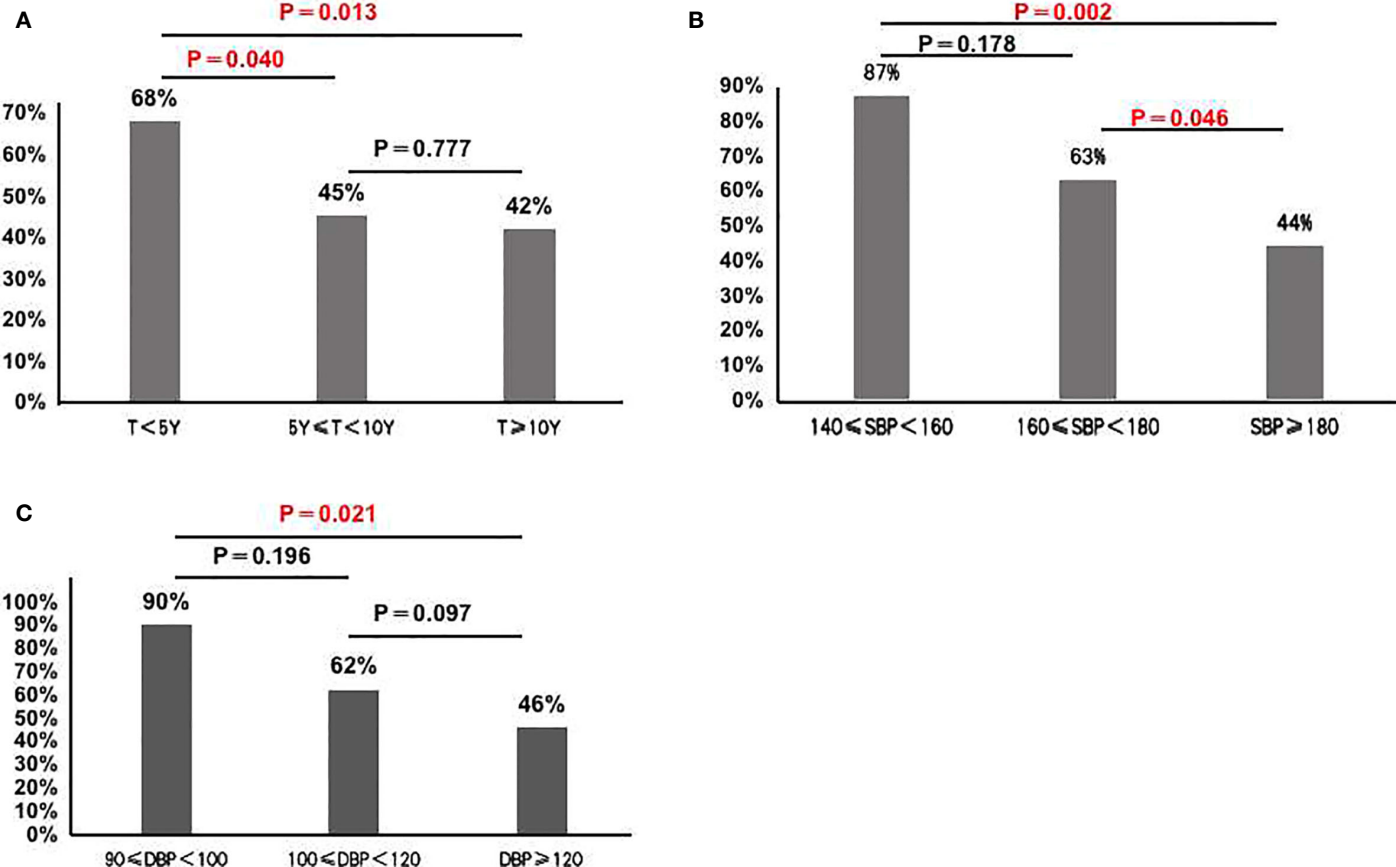
**(A)** Cure rate of hypertension in the group with hypertension during less than 5 years, more than 5 years and 10 years (68% *vs.* 45% *vs* 42%); **(B)** Cure rate of hypertension in the group with SBP<160 mmHg group, 160 mmHg≤SBP<180 mmHg group and SBP≥180 mmHg group (87% *vs*. 63% *vs* 44%); **(C)** Cure rate of hypertension in the group with DBP<100mmHg group, 100mmHg≤DBP < 120mmHg group and DBP≥120mmHg group(90% *vs*. 62% *vs*. 46%).

In 130 classical patients (including APA and APN), 70 patients had complete clinical success, and 60 patients had partial clinical success. 38 out of the 60 patients (63%) still needed antihypertensive medication. Compared to those with postoperative hypertension, postoperative normotensive patients had a shorter duration of hypertension, a lower maximal SBP and maximal DBP, a lower computed tomography (CT) value, and a tendency of higher proportion of females (**Table 2** and **Figure 2**).

**Table 2:** Comparison of clinical characteristics between 130 patients with classical histopathologic findings with complete and partial clinical success.

Variable	Complete (N = 70)	Partial (N = 60)	P value
**Age (years)**	44 ± 10	46 ± 10	0.195
**Gender (M/F)**	26/44	31/29	0.096
**Duration of hypertension (months)**	48 (11,120)	72 (39,120)	0.005
**Duration of hypokalemia (months)**	6 (3,22)	9 (4,24)	0.153
**Preoperatively**			
**The highest SBP (mmHg)**	174 ± 18	192 ± 26	<0.001
**The highest DBP (mmHg)**	107 ± 10	117 ± 15	<0.001
**Serum potassium (mmol/L)**	2.5 ± 0.5	2.5 ± 0.5	0.915
**PRA (ng/ml/h)**	0.01 (0.01,0.01)	0.01 (0.01,0.06)	0.446
**PAC (ng/dl)**	19.7 ± 7.0	20.5 ± 6.9	0.535
**LDL (mmol/L)**	2.62 ± 0.70	2.55 ± 0.68	0.600
**Family history of hypertension**	24% (17/70)	17% (10/59)	0.330
**Diabetes**	4% (3/68)	15% (9/59)	0.103
**Smoker**	13% (9/70)	22% (13/59)	0.214
**Cardiac complications**	32% (18/56)	36% (17/47)	0.887
**Renal complication**	0% (0/70)	0% (0/60)	——
**Tumor size (mm)**	15.0 (12.3,20.0)	16.5 (13.0,21.5)	0.780
**Tumor location (Left/Right)**	35/35	37/23	0.182
**CTvalue (HU)**	2.0 (-5.5,5.0)	5.0 (-4.7,8.8)	0.041
**Postoperatively**			
**SBP (mmHg)**	122 ± 9	136 ± 11	<0.001
**DBP (mmHg)**	79 ± 5	89 ± 7	<0.001
**Serum potassium (mmol/L)**	4.3 ± 0.4	4.2 ± 0.3	0.045
**Antihypertensive medications**	0% (0/70)	63% (38/60)	<0.001
**Potassium supplementation**	0% (0/70)	0% (0/60)	——

**Figure 3:**
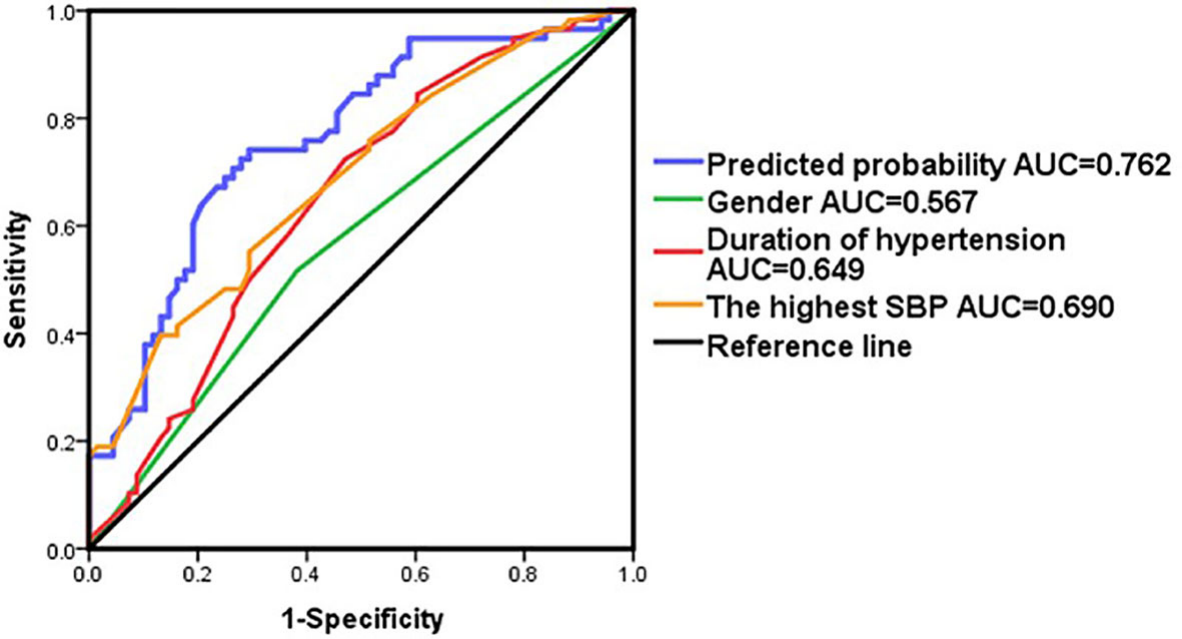
ROC curve in patients by adding variables as gender,duration of hypertension and the highest SBP.

Binary logistic regression analysis revealed that gender, duration of hypertension and the highest SBP were significant independent predictors for the postoperative cure of APA (**Table 3**). A multiple logistic regression model integrating these three predictors was constructed to predict the outcome. AUC of the multiple logistic regression prediction model (0.762) was significantly higher than that of gender, duration of hypertension and the highest SBP alone (P<0.05). The prediction model had a sensitivity of 72.4% and a specificity of 73.1%, and the positive predictive value was 70% and the negative predictive value was 75% (AUC = 0.762; 95% confidence interval = 0.678–0.846) (**Figure 3** and **Supplement 1**).The Hosmer-Lemeshow test indicated that the model fits the data well (P>0.1).

**Table 3:** Estimates of parameters of logistic regression model for complete clinical success of patients with classical histopathologic findings.

Variables	Coefficient	SE	Wald	P-value	OR (95% CI)
**Gender**	1.137	0.435	6.828	0.009	3.118 (1.329 to 7.31)
**Duration of hypertension (month)**	0.006	0.003	4.916	0.027	1.006 (1.001 to 1.01)
**The highest SBP (mmHg)**	0.042	0.011	14.714	0.000	1.043 (1.021 to 1.066)
**Constant**	-8.909	2.104	17.924	0.000	0.000

CI, confidence interval.

## Discussion

In our cohort, based on pathological diagnosis of APA (11), the specificity of CT imaging for APA diagnosis was up to 90%. Although 10% of the patients were preoperatively suspected of APA by CT, and finally diagnosed as APN and NFA based on their pathological characteristics, the blood pressure of all patients in our cohort was relieved, and the cure rate of hypokalemia was 100%. Since all patients in our cohort clinically benefited from the operation, for the PA patients with hypokalemia, if CT reveals a solitary, hypodense adenoma and normal contralateral adrenal morphology, AVS may not be necessary, and surgery can be performed directly. Recently, Burrello et al. proposed the SPACE(Subtyping Primary Aldosteronism by Clinical Evaluation) score for determination of patients that can avoid AVS before surgery. They suggested that patients with a score greater than 16 points can avoid AVS (17). In our study, 117 (85%) patients have a SPACE score greater than 16 points. In fact, a patient with a solitary nodule greater than 1 cm, hypokalemia and normal contralateral adrenal gland should have 16.5 points of SPACE. In our study, patients with NFA with APM or MAPM achieved complete or partial clinical success after adrenalectomy. They would appear to have been not inappropriately treated by unilateral adrenalectomy. Even if the remaining adrenal also has nodules, it may be easier to treat with mineral corticoid receptor antagonists.

In our study, patients with NFA had a longer duration of hypertension than those with APA and APN. They probably had relatively mild symptoms and progress slowly, thus leading to the delay of diagnosis. Patients with APA had a longer duration than those with APN. We speculate that APN may be the precursor of APA, because both APA and APN were dominated by KCNJ5 mutation (unpublished data).

In previous studies, surgical outcomes of APA patients were mainly based on the histopathology of HE staining or AVS instead of functional pathology (7, 12–14, 18, 19). A study from Mayo Clinic observed the outcome of unilateral adrenalectomy in 263 patients with presumptive unilateral disease diagnosed by imaging and AVS. Hypertension was cured in 53patients (41.7%). Whereas 59 patients (46.5%) showed hypertension improvement (12). Recently, CYP11B2 IHC was widely used in studies to examine the functional morphology of normal and pathological adrenals (11, 20) and occasionally in subtyping and outcome prediction in PA (21, 22). Volpe C. et al. examined the clinical outcomes of APA with definite functional, IHC features. In this study, all but one patient (93/94) with APA were biochemically cured after adrenalectomy. Forty-six percent of the APA patients arrived at a complete clinical cure (23). In our study, hypertension was cured in 54% and improved in 46% of the APA patients who were identified based on the new pathological consensus (11).

In our study, the predictive factors of postoperative clinical cure of patients included female gender, shorter duration of hypertension and lower level of blood pressure. Citton et al. also found that female gender, a fewer number of antihypertensive drugs, and a shorter duration of hypertension were the main predictors of hypertension cure (18). In addition, the present study developed a model, integrating multiple predictors to predict the prognosis of surgery, which is easy to use and is more accurate than single clinical parameter for the prediction of surgical outcomes.

### Strengths and Limitations of the Study

The strength of our study lies in that it employed the diagnostic criteria of APA according to HISTALDO consensus, which are superior to pathological examination with only HE staining or AVS alone. Diagnosis by only HE staining cannot exclude NFA, and AVS cannot rule out adrenal nodular hyperplasia with lateralization. In addition, our results support the feasibility to skip AVS in patients with hypokalemic PA, which may simplify the diagnostic procedures and bring benefits to patients.

However, our study had some limitations: (1) Because postoperative data on ALD and PRA were not available in most patients, only clinical parameters were used in the outcome evaluation. Furthermore, lack of AVS for PA classification is another issue. (2) The present paper focused only on the patients with hypokalemia, the severe kind of PA. The PA patients with normokalemia or APA without typical features on CT were excluded, though these patients account for a certain proportion in daily practice. Therefore, our conclusion may not be extrapolated to all PA patients. It is speculated that there will be larger proportion of nodular hyperplasia and NFA in the patients without hypokalemia. (3) The limited number of APN and APM or MAMP is statistically relevant for the comparison. And the limited number of APN is likely due to the fact that indication for surgery was made on the basis of CT alone, and not AVS.(4) The prediction model for clinical outcomes established in our study needs to be verified by a validation cohort.

## Conclusion

The specificity of CT for the diagnosis of classical PA (APA and APN) with hypokalemia based on functional pathology was 95%. Practically all patients achieved complete or partial clinical success after operation. Gender, duration of hypertension and the highest SBP were independent predictors for the postoperative cure of APA.

## Data Availability Statement

The datasets presented in this study can be found in online repositories. The names of the repository/repositories and accession number(s) can be found below: Genome Sequence Archive in National Genomics Data Center, Beijing Institute of Genomics (China National Center for Bioinformation), Chinese Academy of Sciences, under accession number HRA000965.

## Ethics Statement

All procedures performed in studies involving human participants meet the ethical standards of the institutional research committee (Ethics committee of Peking Union Medical College Hospital; S-K431). The patients/participants provided their written informed consent to participate in this study.

## Author Contributions

Conceived and designed the experiments: AT and YL. Collected and analyzed clinical data: HW, FW, YZ, JW, DD, SC, LL, WR. Conducted radiological and pathological analysis: XC, HS, XM, YC. Write the manuscript: HW, FW. Revise the manuscript: AT. All authors contributed to the article and approved the submitted version.

## Funding

This research was supported by National Natural Science Foundation of China (Grant No. 81770427, and 82070822) and CAMS Innovation Fund for Medical Sciences(CIFMS) of China (Grant No. 2017-I2M-1-001).

## Conflict of Interest

The authors declare that the research was conducted in the absence of any commercial or financial relationships that could be construed as a potential conflict of interest.

## Publisher’s Note

All claims expressed in this article are solely those of the authors and do not necessarily represent those of their affiliated organizations, or those of the publisher, the editors and the reviewers. Any product that may be evaluated in this article, or claim that may be made by its manufacturer, is not guaranteed or endorsed by the publisher.
