# Supplementary material for: Surgical Outcomes of Aldosterone-Producing Adenoma on the Basis of the Histopathological Findings
Source: Front Endocrinol (Lausanne). 2021 Sep 6;12:663096. doi: 10.3389/fendo.2021.663096 (PMC8451176; doi:10.3389/fendo.2021.663096)
Supplement: Supplementary file 1 [file Presentation_1.pdf]

Supplement 1

Mathematically,themultiple logistic regression model is as follows:

Logit(P)= -8.909 + 1.137*sex(male=1; female=0) + 0.006*duration of hypertension(months) +0.042*SBP(mmHg)

$$P = \frac{e^{\mathrm{Logit(P)}}}{1 + e^{\mathrm{Logit(P)}}}$$

where P represents prediction probability. Prediction of the outcome could be conducted according to the condition of the patient, including gender, duration of hypertension and the highest SBP, and the prediction probability.

Predictedprobability	Sensitivity	Specificity	Sensitivity+ Specificity
0	1	0	1
0.0714635	1	0.015	1.015
0.0882071	1	0.029	1.029
0.097739	1	0.044	1.044
0.103437	0.983	0.044	1.027
0.1061573	0.983	0.059	1.042
0.1076819	0.966	0.059	1.025
0.1144617	0.966	0.074	1.04
0.1244408	0.966	0.088	1.054
0.1410636	0.966	0.103	1.069
0.1550572	0.966	0.118	1.084
0.1567291	0.966	0.132	1.098
0.1647549	0.966	0.147	1.113
0.1740214	0.966	0.162	1.128
0.1766395	0.948	0.162	1.11
0.180867	0.948	0.176	1.124
0.1854847	0.948	0.191	1.139
0.1910456	0.948	0.206	1.154
0.1981733	0.948	0.221	1.169
0.2046523	0.948	0.25	1.198
0.2118256	0.948	0.265	1.213
0.2162609	0.948	0.279	1.227
0.2173435	0.948	0.294	1.242
0.2180989	0.948	0.309	1.257
0.2197376	0.948	0.324	1.272
0.2238881	0.948	0.338	1.286
0.230436	0.948	0.353	1.301
0.2399031	0.948	0.368	1.316
0.2510367	0.948	0.382	1.33
0.2651404	0.948	0.397	1.345
0.2748232	0.948	0.412	1.36

0.2769167	0.931	0.412	1.343
0.2796992	0.914	0.412	1.326
0.2822729	0.914	0.426	1.34
0.2841613	0.897	0.441	1.338
0.2856475	0.879	0.441	1.32
0.2975983	0.879	0.456	1.335
0.3100309	0.879	0.471	1.35
0.3144001	0.862	0.471	1.333
0.3180903	0.862	0.485	1.347
0.3250986	0.845	0.485	1.33
0.3359308	0.845	0.5	1.345
0.3445201	0.845	0.515	1.36
0.3548867	0.81	0.544	1.354
0.3653751	0.793	0.544	1.337
0.3818089	0.776	0.544	1.32
0.3938706	0.776	0.559	1.335
0.3959148	0.759	0.574	1.333
0.4002565	0.759	0.603	1.362
0.4051006	0.741	0.603	1.344
0.4105155	0.741	0.618	1.359
0.4224609	0.741	0.632	1.373
0.4356932	0.741	0.647	1.388
0.4419471	0.741	0.662	1.403
0.4509768	0.741	0.676	1.417
0.4636763	0.741	0.691	1.432
0.470941	0.741	0.706	1.447
0.4754322	0.724	0.706	1.43
0.4895113	0.724	0.721	1.445
0.5059285	0.707	0.721	1.428
0.5141714	0.707	0.735	1.442
0.5186954	0.69	0.735	1.425
0.5277261	0.69	0.75	1.44
0.5355086	0.672	0.75	1.422
0.5360132	0.672	0.765	1.437
0.5382645	0.638	0.794	1.432
0.5402669	0.603	0.809	1.412
0.5429505	0.586	0.809	1.395
0.5504304	0.569	0.809	1.378
0.5567156	0.517	0.809	1.326
0.5585167	0.517	0.824	1.341
0.5639702	0.5	0.824	1.324
0.569354	0.5	0.838	1.338
0.5720021	0.483	0.838	1.321
0.5760022	0.466	0.853	1.319

0.5809116	0.431	0.853	1.284
0.5937325	0.431	0.868	1.299
0.6179949	0.414	0.868	1.282
0.6387794	0.397	0.868	1.265
0.6459138	0.397	0.882	1.279
0.6514056	0.379	0.882	1.261
0.6616682	0.379	0.897	1.276
0.6676023	0.362	0.897	1.259
0.6817956	0.345	0.897	1.242
0.6985056	0.328	0.897	1.225
0.7031013	0.31	0.897	1.207
0.7150413	0.293	0.897	1.19
0.7268915	0.259	0.897	1.156
0.7364527	0.259	0.926	1.185
0.7452707	0.241	0.926	1.167
0.7494393	0.207	0.956	1.163
0.7588695	0.19	0.956	1.146
0.7812784	0.172	0.956	1.128
0.8037772	0.172	0.985	1.157
0.8158748	0.172	1	1.172
0.8394656	0.155	1	1.155
0.8686609	0.121	1	1.121
0.8926006	0.103	1	1.103
0.9120624	0.086	1	1.086
0.9274809	0.052	1	1.052
0.9375156	0.034	1	1.034
0.9562734	0.017	1	1.017
1	0	1	1
